# The role of microRNAs in primary Sjögren’s disease: deciphering regulatory networks and assessing current therapeutic perspectives

**DOI:** 10.3389/fimmu.2025.1669382

**Published:** 2025-10-16

**Authors:** Wang Chengzhi, Li Songwei, Liu Yifan, Du Mengmeng, Li Huan

**Affiliations:** ^1^ The Rheumatology Department of the First Affiliated Hospital of Henan University of Chinese Medicine, Zhengzhou, China; ^2^ The First Clinical College of Henan University of Chinese Medicine, Zhengzhou, China; ^3^ The Second Affiliated Hospital of Henan University of Chinese Medicine, Zhengzhou, China

**Keywords:** microRNA, primary Sjögren’s disease, regulatory networks, therapeutic perspectives, macrophage polarization, apoptosis, Th17/Treg balance

## Abstract

Primary Sjögren’s disease (SjD) is a chronic systemic autoimmune disorder whose pathogenesis remains incompletely understood. Current clinical interventions demonstrate limited efficacy, yielding suboptimal therapeutic outcomes. microRNAs (miRNAs)–critical regulators of transcriptional networks–participate in SjD pathogenesis through multifaceted mechanisms. Dysregulated miRNA expression during SjD progression directly influences disease prognosis, establishing miRNAs as promising therapeutic targets. Evidence implicates macrophage polarization, apoptosis dysregulation, Th17/Treg imbalance, T/B lymphocyte dysfunction, glandular impairment, and aberrant type I interferon responses in SjD development. Notably, miR-216a-3p, miR-31-5p, and miR-155-5p modulate key signaling pathways (NF-κB, JAK/STAT, PI3K/AKT) to optimize macrophage polarization, suppress apoptosis, restore Th17/Treg equilibrium, regulate T/B lymphocyte activity, enhance glandular function, normalize type I interferon responses,thereby exerting potent anti-SjD effects. This review synthesizes recent literature to elucidate SjD pathogenesis and miRNA-mediated therapeutic mechanisms, providing a theoretical foundation for novel SjD management strategies.

Primary Sjögren’s disease (SjD) is a chronic autoimmune disease characterized by exocrine gland dysfunction, leading to symptoms such as dry mouth and dry eyes that significantly impair patients’ quality of life, and severe complications may even be life-threatening (1, 2). The pathogenesis of SjD is highly complex, involving multiple factors such as genetics, environment, host factors, and immune function; however, the exact mechanisms remain elusive (3). In SjD, lymphocyte infiltration occurs in the salivary and lacrimal glands. During early disease stages, CD4^+^ T cell infiltration predominates; as the disease progresses, B cell infiltration increases and becomes more prominent (4). Currently, there are still no disease-modifying drugs specifically for SjD. Treatment primarily aims to relieve clinical symptoms; however, drug-related adverse effects often limit its overall efficacy (5). Consequently, there is an urgent need to develop safer and more effective therapies.

## 1 Overview of microRNA

microRNA (miRNA) is an endogenous, non-protein-coding RNA, primarily transcribed in the nucleus, with a length of approximately 20–22 nucleotides. Functionally, miRNA regulates gene expression post-transcriptionally, primarily through gene silencing, and plays a significant role in modulating disease-related genes (6). Through diverse molecular pathways, miRNA exerts regulatory effects on critical physiological and pathological processes, including cell growth, development, differentiation, proliferation, and apoptosis (7). Mature miRNA is released into the receptor cells to participate in transcription and gene expression, and simultaneously regulates various cellular and molecular pathways (8).

### 1.1 The biological occurrence process of miRNA

miRNA regulates gene expression by targeting the 3’ untranslated region (3’ UTR) of mRNA. Its biogenesis involves tightly regulated steps, starting with transcription by RNA polymerase II or III. The resulting primary transcript (pri-miRNA) is processed through either classical or non-classical pathways (9). In the classical pathway, pri-miRNA is cleaved by a complex containing DGCR8 and the RNase III enzyme Drosha. DGCR8 recognizes specific motifs such as “GGAC” and m6A modifications in pri-miRNA (10). Drosha then trims the pri-miRNA into a precursor miRNA (pre-miRNA) with a 2–3 nucleotide 3’ overhang (11). Pre-miRNA is exported to the cytoplasm via exportin-5/RanGTP and further processed by Dicer, which removes the terminal loop to produce a miRNA duplex (12). One strand (5p or 3p) is selected as the guide strand based on 5’ end stability or the presence of a 5’ uridine, and loaded into an AGO protein (AGO1–4 in humans) (13, 14). The passenger strand is typically degraded-cleaved by AGO2 if perfectly complementary, or passively removed if mismatched (15). Non-classical pathways enhance the diversity of miRNA regulation and rely on alternative combinations of processing factors (16). These include DGCR8/Drosha-independent routes, such as Mirtrons (derived from introns) and m7G-capped pre-miRNAs that are exported via exportin-1 without Drosha cleavage (17). Dicer-independent pathways involve shRNA-derived pre-miRNAs processed by Drosha and matured in the cytoplasm by AGO2, where the 3’ strand is trimmed to facilitate 5’ strand maturation (18).

### 1.2 The mechanism of miRNA transcriptional action

The transcriptional regulatory mechanism of miRNA mainly includes two ways: translation inhibition and mRNA degradation. These two mechanisms can work together and are closely related to the degree of complementarity between miRNA and its target (19). In animal cells, translation inhibition is the most important and widely existing mechanism: when miRNA binds to the 3’ untranslated region (3’ UTR) of the target mRNA but does not have a complete complementary match, it mainly inhibits the translation process (20). while when the degree of complementarity is higher, it causes mRNA degradation (21). This regulatory mechanism enables cells to rapidly and flexibly adjust the protein composition without changing the DNA sequence of the gene and the quantity of the transcript, in response to developmental signals, environmental pressures, and various physiological and pathological processes (22).

In conclusion, miRNA plays a pivotal role in regulating immune responses and its dysregulation is implicated in metabolic disorders, tumors, and autoimmune diseases (23). miRNA plays a crucial role in almost all cellular activities and can exert biological functions through multiple pathways (24). By specifically binding to target genes, miRNA regulates critical processes such as inflammatory responses, macrophage polarization, and cell apoptosis. Consequently, miRNA represents a major focus in biological research and an emerging therapeutic target for numerous diseases.

## 2 Future treatment strategies for SjD based on miRNA

**Figure 1:**
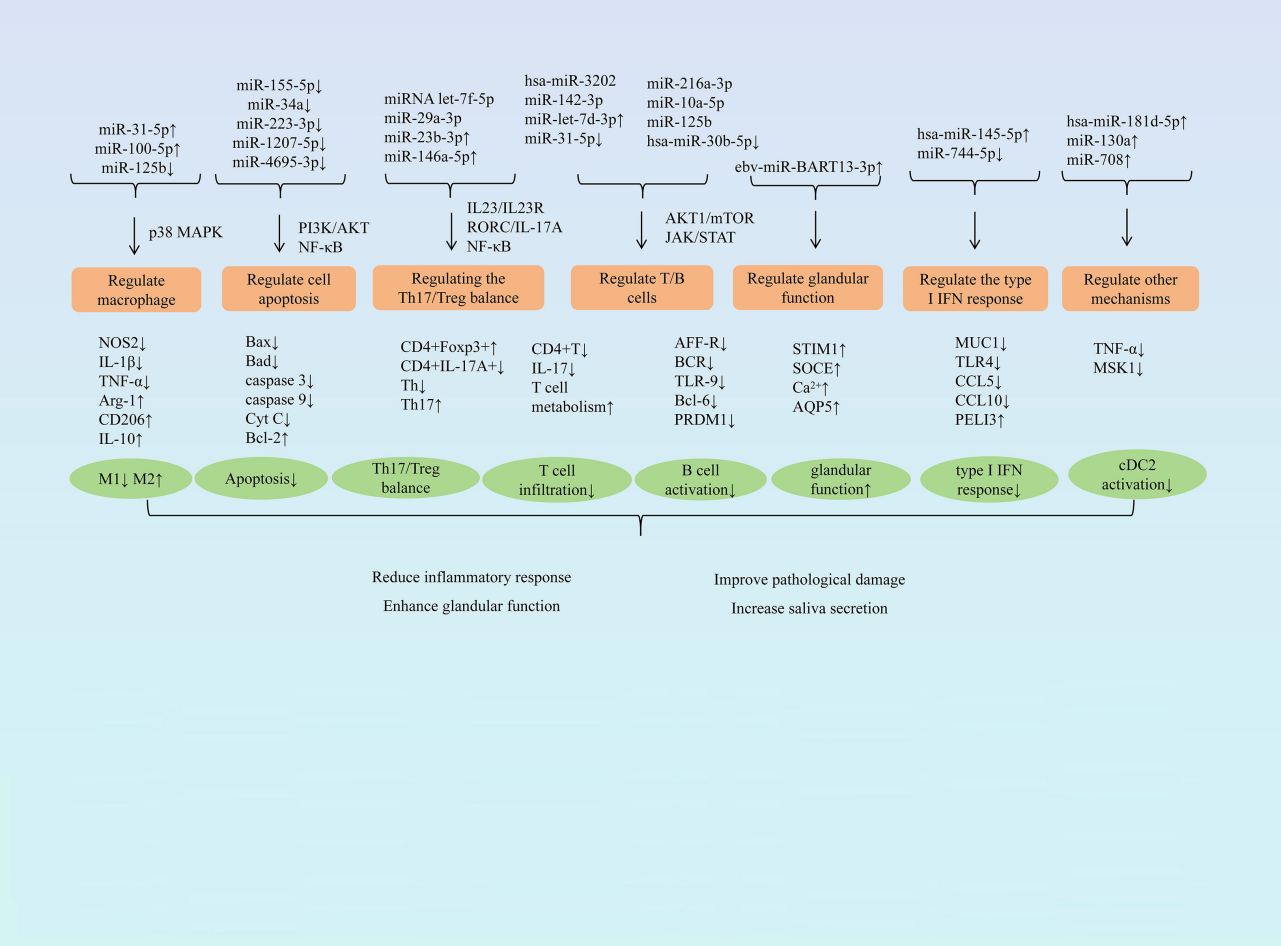
Summary of the mechanism of miRNA treatment for SjD.

Research has demonstrated that miRNA exerts protective effects against SjD through multiple pathways, including modulating macrophage polarization, influencing cell apoptosis, balancing the Th17/Treg ratio, regulating T and B lymphocyte activity, restoring glandular function, and regulating type I IFN responses. (**Figure 1** summarizes the mechanism of miRNA in treating SjD).

### 2.1 miRNA regulation of macrophage polarization

As key components of the innate immune system, macrophages reside in all tissues and organs, performing critical functions including immune regulation, tissue repair, and maintaining immune homeostasis (25). Dysfunctional macrophages contribute to impaired tissue repair/regeneration and excessive inflammatory factor production in various diseases (26–28). Conversely, M2 macrophages, activated by IL-4 or IL-13, secrete anti-inflammatory cytokines (e.g., IL-10, TGF-β) which generally suppress inflammation; however, sustained M2 activity can promote the progression of certain chronic inflammatory conditions (29, 30). In early SjD, M1 macrophage polarization increases, accompanied by elevated secretion of inflammatory factors (TNF-α, IL-6, IL-1β, IL-12), exacerbating submandibular gland inflammation (31). As SjD progresses, chronic inflammation transitions to irreversible salivary gland fibrosis, a process predominantly mediated by M2 macrophages (32). This imbalance, characterized by upregulated M1 and downregulated M2 polarization, is a hallmark of SjD (33). Targeting this imbalance offers therapeutic potential. Myeloid-derived growth factor (MYDGF) alleviates SjD symptoms and improves submandibular gland function by inhibiting M1 macrophage infiltration and promoting M2 polarization via suppression of the CX3CL1/CX3CR1 axis (34). Therefore, modulating macrophage polarization represents a significant strategy for ameliorating SjD.

**Figure 2:**
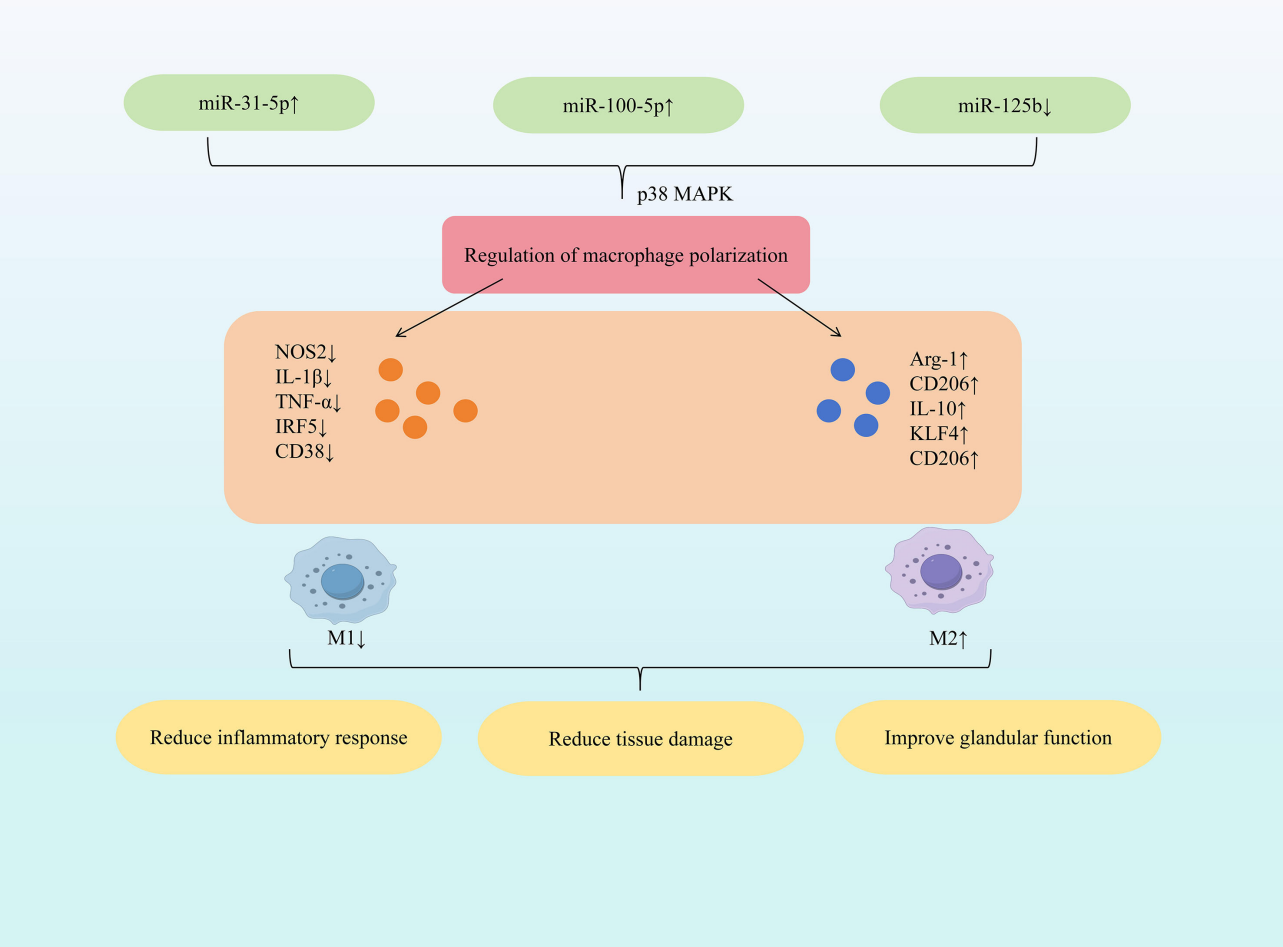
Mechanisms of miRNA-mediated macrophage polarization in SjD therapy.

Research indicates that specific miRNAs (e.g., increased miR-31-5p, miR-100-5p; decreased miR-125b) can regulate macrophage cytokine and marker expression. This promotes polarization towards the anti-inflammatory M2 phenotype, dampens inflammatory responses, and modulates Th17/Treg balance, ultimately exerting protective effects against SjD. Among all the modifications, N6-methyladenosine (m6A) is the one that has been most extensively studied and is the most commonly found internal chemical modification on mRNA and non-coding RNA (35). m6A is a key upstream regulatory factor in miRNA biogenesis, capable of modifying various components in the miRNA generation pathway. It regulates RNA transcription, translation and stability, thereby widely influencing the level and function of miRNA (36). Dysregulation of m6A regulatory factors is implicated in the pathogenesis of Sjögren’s syndrome (SS) (37, 38). Study demonstrated that increased expression of m6A-modified miR-31-5p directly targets the P2X7 receptor, downregulating p38 MAPK expression and inhibiting the p38 MAPK signaling pathway. This cascade reduces NOS2, IL-1β, and TNF-α expression while increasing Arg-1, CD206, and IL-10 expression. Consequently, M1 macrophage activation is suppressed, M2 macrophage activation is promoted, restoring the M1/M2 balance. This shift alleviates inflammatory cell infiltration and tissue damage, thereby reducing dry eye symptoms (39). Mesenchymal stem cells (MSCs), pluripotent stem cells isolated from bone marrow and other tissues, primarily promote regeneration and modulate immune responses via paracrine mechanisms (40). Extracellular vesicles (EVs) derived from early-passage induced MSCs (iMSCs) effectively inhibited salivary gland inflammation onset in SS mouse models, with efficiency comparable to EVs from young iMSCs and bone marrow MSCs (41). Further research demonstrated that inhibiting miR-125b expression in senescence-associated iMSC-EVs reduced CD38 expression and increased CD206 expression, lowering the CD38/CD206 ratio and promoting M2 macrophage polarization. This intervention also reduced the expression of IL-17a and IL-21, inhibited the differentiation of Th17 cells, and prevented the occurrence of salivary gland inflammation (42). Mesenchymal stem cell-derived small extracellular vesicles (MSC-sEVs) exhibit potent immunomodulatory properties and have therapeutic potential for various autoimmune diseases (43). Specifically, miR-100-5p within human umbilical cord MSC-sEVs (hUC-MSC-sEVs) exerts significant anti-SS effects. Elevating miR-100-5p expression reduced NOS2 and IRF5 expression while increasing CD206, Arg-1, and KLF4 expression, promoting M2 macrophage polarization. Furthermore, it increased the proportion of CD4^+^Foxp3^+^Tregs and promoted Treg differentiation. These changes decreased TNF-α, IL-1β, and IL-6 expression, mitigating inflammation. Consequently, this approach alleviated dry eye disease severity and ameliorated inflammation in both the tear film and conjunctiva (44). (**Figure 2** illustrates miRNA-mediated macrophage polarization mechanisms in SjD therapy).

### 2.2 miRNA modulation of cell apoptosis

Apoptosis, an autonomous and orderly programmed cell death process, is essential for organismal development and tissue homeostasis (45). It is primarily regulated through endogenous and exogenous pathways. The endogenous pathway is chiefly controlled by Bcl-2 family proteins, with Bax and Bcl-2 playing pivotal roles as key components (46). The caspase family of highly conserved cysteine proteases also significantly contributes to apoptosis (47). Exogenous pathways are typically activated by death receptors or Toll-like receptors (TLRs), leading to receptor-interacting protein kinase 1 formation, caspase cascade initiation, and subsequent apoptosis (48). When immune function is impaired, apoptosis protects tissues from damage; dysregulation of this process may contribute to SjD pathogenesis (15). Apoptosis of exocrine gland cells represents a key early pathological change in SjD, promoting innate immune system activation and lymphocyte-mediated autoimmune responses (49). Specific apoptotic factors further participate in submandibular gland dysfunction in SjD (50). Exogenous and endogenous apoptotic pathways can be involved in the entire process of the occurrence and development of SjD (51, 52). Consequently, modulating apoptosis may alleviate immune-inflammatory responses and delay SjD advancement.

**Figure 3:**
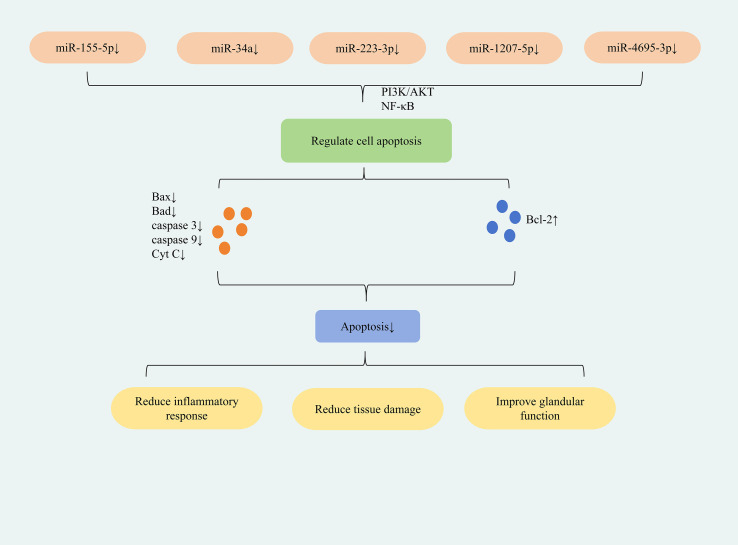
Mechanisms of miRNA-mediated regulation of apoptosis in SjD therapy.

The study found that down-regulation of miR-155-5p expression can inhibit apoptosis in HSGEC and SGECs cells; down-regulation of miR-34a expression can inhibit apoptosis in HSGE cells; down-regulation of miR-223-3p expression can inhibit apoptosis in SEGCs cells; down-regulation of miR-1207-5p and miR-4695-3p expression can inhibit apoptosis in HSG cells. This reduces inflammation, restores glandular secretion, and ultimately ameliorates SjD symptoms. The differences in the roles of specific miRNAs in different tissues or cells mainly result from mechanisms such as the cell type specificity of target gene expression, the diversity of signaling pathway networks, and the different binding affinities between miRNAs and their targets. Located on human chromosome 21, miR-155 is a well-characterized miRNA that regulates immune cell development, differentiation, and function (e.g., in B cells, T cells, and dendritic cells), playing critical roles in immune-inflammatory responses (53). Downregulation of miR-155-5p was found to upregulate PIK3R1 expression, lower the p-PI3K/PI3K and p-AKT/AKT ratios, and thereby inhibit the PI3K/AKT signaling pathway. This suppressed apoptosis while promoting proliferation, viability, and colony formation in SjD submandibular gland epithelial cells (HSGECs). Concomitantly, it elevated anti-inflammatory cytokines (IL-10, IL-4and reduced pro-inflammatory cytokines (TNF-α, IL-6), mitigating inflammation. Ultimately, this restored glandular secretory function and ameliorated symptoms in SjD mice (54). miR-155-5p exhibits multifaceted functions, including regulation of tumorigenesis, immunomodulation, and oxidative stress (55, 56). Its immunoregulatory role further implicates it in multiple immune-related disorders, including SS (57). ARRB2 functions as a downstream target gene of miR-155-5p. The study demonstrated that downregulation of miR-155-5p expression directly targets ARRB2, leading to reduced expression of NF-κB p65 and a decreased phospho-IκBα-to-IκBα ratio. This consequently suppresses the NF-κB signaling pathway. This inhibition decreased the expression of pro-apoptotic proteins Bax, caspase-3, and caspase-9 while concomitantly increasing anti-apoptotic Bcl-2 expression, ultimately suppressing apoptosis in SGEC cells. Furthermore, NF-κB pathway suppression lowered TNF-α and IL-6 expression, mitigating the inflammatory response and reducing salivary gland damage (58). miR-34a is implicated in the development of various inflammatory diseases and is upregulated in the tears of patients with SS (59). In patients with severe SjD, NF-κB p65 expression was significantly elevated, whereas IκBα expression was reduced, collectively indicating activation of the NF-κB signaling pathway. Concurrently, key pro-apoptotic factors associated with the mitochondrial pathway—including pro-caspase-9, caspase-9, cleaved-caspase-3, and Cyt C—were markedly upregulated. This upregulation signifies mitochondrial damage and activation of apoptotic responses within the salivary glands. Downregulation of miR-34a expression effectively suppressed NF-κB pathway activation, upregulated Bcl-2 expression, and downregulated pro-apoptotic factors including Bad, pro-caspase-9, caspase-9, cleaved-caspase-3, and cytochrome c. Collectively, these changes inhibited the mitochondrial apoptosis pathway and alleviated salivary gland damage in NOD mice (60). ITPR3 is a direct downstream target of miR-223-3p and is negatively regulated by it. In samples from SS patients and in IFNγ-induced submandibular gland epithelial cells (SGECs), miR-223-3p expression was upregulated, whereas ITPR3 expression was downregulated.The study demonstrated that inhibiting miR-223-3p expression reduced Bax and caspase-3 levels while elevating Bcl-2 expression via the NF-κB pathway, thereby suppressing apoptosis in SGECs. This inhibition also decreased the expression of pro-inflammatory cytokines IL-6, IL-12, and TNF-α, thus mitigating inflammatory responses. Furthermore, this intervention enhanced SGEC viability under IFNγ stimulation and ultimately alleviated ocular dryness in SS patients (61). Through miRNA analysis, two miRNAs targeting TRIM21 were identified, namely miR-1207-5p and miR-4695-3p (62). These two miRNAs mainly exerted their effects through the apoptotic pathway and were also specific anti-apoptotic miRNAs. Further research demonstrated that downregulating miR-1207-5p and miR-4695-3p expression could reduce Bax, CASP-9, and CASP-8 expression and increased Bcl-2 expression. This suppressed apoptosis in HSG cells, improved salivary gland function, and enhanced saliva secretion in SS patients (63). (**Figure 3** depicts miRNA-regulated apoptotic mechanism for SjD treatment).

### 2.3 miRNA maintenance of Th17/Treg balance

Helper T cell 17 (Th17) and regulatory T cells (Treg) play key roles in the pathogenesis of various autoimmune diseases (64). Transforming growth factor-β (TGF-β) is a pivotal regulator of the interaction and balance between Th17 and Treg cells. TGF-β induces the expression of the lineage-defining transcription factors retinoic acid receptor-related orphan receptor γt (RORγt) and forkhead box P3 (Foxp3), primarily by activating signal transducer and activator of transcription 3 (STAT3) (65). Th17 cells can promote inflammatory responses by secreting pro-inflammatory factors such as IL-17A, IL-17F, and IL-21 (66). Treg cells are a type of negatively regulatory cells that can alleviate inflammatory responses by releasing anti-inflammatory factors such as IL-10 (67). Th17/Treg immune imbalance contributes to glandular damage in SjD. Platelet-derived peptides suppress Th17 cell activation, promote Treg differentiation, restore Th17/Treg balance, and thereby ameliorate SS-related dry eye syndrome and autoimmune inflammation (68). In SS model mice, elevated Th17 cells and IL-17 levels were observed in exocrine glands, accompanied by significant reductions in Treg cells and TGF-β (69). Targeted downregulation of IL-6, TNF-α, and IL-17 reduced the Th17/Treg ratio, expanded Treg cell populations, enhanced Treg functionality, and thereby restored immune balance. This rebalancing significantly attenuated salivary gland inflammation (70).Accordingly, restoring and maintaining the Th17/Treg immune balance is a key therapeutic measure for SjD.

**Figure 4:**
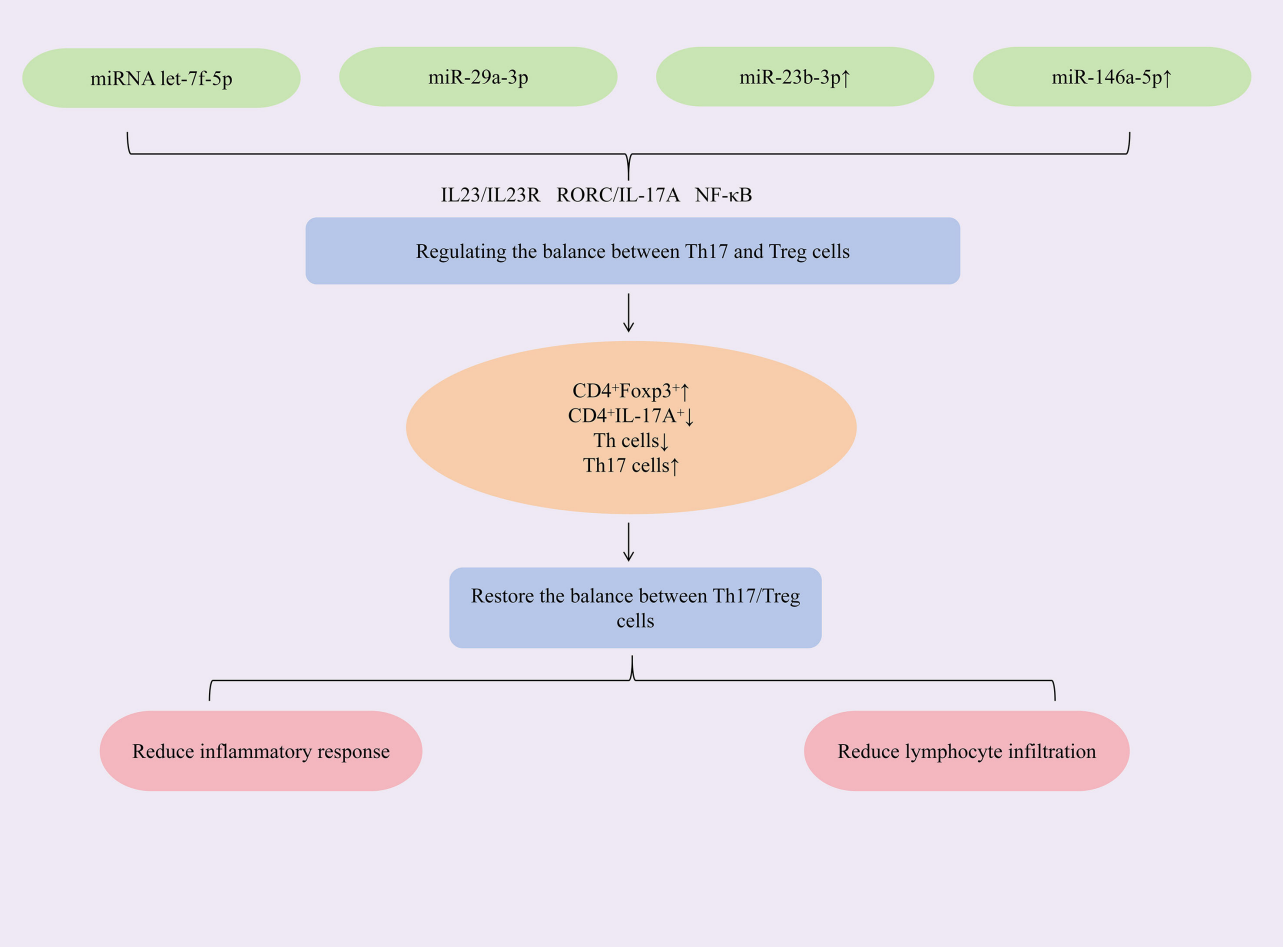
Mechanisms of miRNA modulation of Th17/Treg balance for SjD treatment.

Researches have found that increasing the expression of miRNAs such as let-7f-5p, miR-29a-3p, miR-23b-3p, and miR-146a-5p could restore the Th17/Treg immune balance, alleviate immune-inflammatory responses, and act synergistically to inhibit cell apoptosis and reduce CD4^+^ T lymphocyte infiltration, thereby exerting therapeutic effects against SjD. Mesenchymal stem cells (MSCs) and their extracellular vesicles (EVs) exhibit potent immunomodulatory properties and hold significant therapeutic potential for autoimmune diseases (71, 72). To augment the efficacy of labial gland MSCs (LGMSCs) and their EVs (LGMSC-EVs), researchers loaded exogenous miRNA let-7f-5p into LGMSC-EVs. The study revealed that miRNA let-7f-5p encapsulated in LGMSC-EVs could suppress the RORγt/IL-17A signaling axis. This increased the proportion of CD4^+^Foxp3^+^Treg cells while reduced the proportion of CD4^+^IL-17A^+^Th17 cells, thereby lowering the Th17/Treg ratio and restoring Th17/Treg immune balance. Additionally, it elevated IL-10 and TGF-β expression, reduced IL-6 and IL-17A expression, mitigated inflammation, increased salivary flow rate, and alleviated lymphocytic infiltration in submandibular glands of NOD/ShiLtJ mice (73). Exosomes, natural nanoparticles derived from the endosomal system, demonstrate therapeutic potential in Sjögren’s syndrome. Specifically, human deciduous tooth stem cell-derived exosomes (SHAp-exos) inhibit inflammation-induced epithelial barrier disruption and modulate apoptotic responses through suppression of p-ERK1/2 activation. This dual action consequently enhances salivary secretion and ameliorates SS symptoms (74, 75). The study demonstrated that SHED-exo nanoparticles modulate the miR-29a-3p/T-bet axis, reducing Th1 cell polarization and inhibiting T helper cell differentiation. This attenuated Th1/Th2/Th17-mediated immune-inflammatory responses and restored the glandular immune microenvironment. Critically, treatment decreased CD4^+^ T-cell infiltration in submandibular glands of NOD mice, suppressed pro-inflammatory cytokines (IFN-γ, TNF-α), alleviated inflammation, and enhanced saliva secretion (76). Dysregulated miRNA expression is closely associated with Sjögren’s syndrome pathogenesis. Specifically, miR-23b-3p expression is significantly reduced in the NOD mouse model of SS, and this miRNA demonstrates broad protective effects against multiple autoimmune disorders (77). The study demonstrated that upregulating miR-23b-3p expression targets SOX6, downregulating NF-κB p65 expression and reducing the phospho-IκBα/IκBα ratio, thereby inhibiting the NF-κB signaling pathway. This cascade suppressed pro-inflammatory cytokines (TNF-α, IL-6, IL-17A, IFN-γ) while elevating TGF-β1 expression, rebalancing the Th1/Th17/Treg immune equilibrium. Concurrently, it reduced pro-apoptotic Bax and cleaved caspase-3 levels, increased anti-apoptotic Bcl-2 expression, and attenuated submandibular gland epithelial cell apoptosis—ultimately mitigating glandular inflammation and restoring salivary flow in NOD mice (78). miR-146a-5p is significantly upregulated in peripheral blood mononuclear cells (PBMCs) of primary Sjögren’s syndrome (SjD) patients (79, 80), with meta-analyses supporting its utility as a diagnostic biomarker (81). Mechanistically, upregulation of miR-146a-5p reduces ADAM17 protein levels, thereby decreasing membrane-bound IL-23R (mIL-23R) shedding and activating the IL-23/IL-23R signaling pathway. This cascade elevates IL-17A and IL-21 production, expands the Th17 cell population, enhances Th17 differentiation, and ultimately mitigates immune inflammation in SjD (82). (**Figure 4** demonstrates miRNA modulation of Th17/Treg balance in SjD therapy).

### 2.4 miRNA regulation of T and B lymphocytes

T and B lymphocytes—key effectors of the adaptive immune system—play central roles in autoimmune pathogenesis. Specifically, T lymphocytes orchestrate both pro-inflammatory and immunoregulatory responses, with T-cell-mediated autoimmunity recognized as a critical driver of SjD pathology (83). Patients with SjD exhibit significant infiltration of CD4^+^ and CD8^+^ T lymphocytes in salivary and lacrimal glands. Additionally, CD8^+^ T lymphocytes show abnormal activation and proliferation both in peripheral circulation and around acinar epithelial cells (84). Follicular helper T cells (Tfh) promote B lymphocyte responses within ectopic germinal centers (GC) by secreting IL-21, thereby facilitating B cell activation and differentiation. This process disrupts the formation and function of GC-like structures in the salivary glands of SjD patients (85). In patients with SjD, peripheral B lymphocytes undergo significant proliferation, while substantial B lymphocyte infiltration occurs in salivary gland tissue. This aberrant B cell activity drives excessive autoantibody secretion, contributing to autoimmune pathology (86). Marginal zone B cells (MZB) contribute to the pathogenesis of SjD. In BAFF transgenic mice, reduced MZB cell numbers alleviate SjD manifestations: preserved salivary function, absence of autoantibodies, and normalized histology in salivary and lacrimal glands (87). B-cell activating factor (BAFF) promotes the formation of ectopic GC-like structures and shows elevated serum expression in SjD patients. This elevation positively correlates with anti-Ro/SSA and anti-La/SSB antibody titer (88). Therefore, both T lymphocytes and B lymphocytes critically contribute to the pathogenesis and progression of SjD, directly impacting its treatment and prognosis.

Studies have shown that hsa-miR-3202 can inhibit the proliferation and invasion of Jurkat cells, miR-142-3p promotes T cell activation, upregulation of miR-let-7d-3p expression can reduce the proportion of CD4^+^ T cells, and downregulation of miR-31-5p expression can promote glycolysis in CD4^+^ T cells, inducing an autoimmune T cell response, ultimately exerting an anti-SjD effect. MMP2 is highly expressed in PBMCs and salivary gland tissues of patients with SjD. The study found that hsa-miR-3202 can reduce the expression of MMP2 in Jurkat cells, inhibit T lymphocyte infiltration, improve immune inflammatory responses; and inhibit the proliferation and invasion of Jurkat cells, alleviate salivary gland tissue damage, improve gland function, and increase saliva secretion (89). T-cell exosome-derived miR-142-3p functions as a pathogenic driver of SS immunopathology. This miRNA exhibits predominant expression within immune compartments and critically regulates T-cell functionality (90, 91). Key predictive targets of miR-142-3p include adenylate cyclase 9 (AC9), sarcoplasmic reticulum Ca²^+^-ATPase 2b (SERCA2b), and ryanodine receptor 2 (RyR2)–all essential regulators of salivary gland secretion (92). miR-142-3p impairs salivary gland function through multiple mechanisms: restricting cAMP production, altering calcium signaling, and reducing acinar protein secretion. Concurrently, it promotes T-cell activation and directly disrupts epithelial cell function, collectively exacerbating glandular dysfunction in SS patients. Consequently, targeted reduction of miR-142-3p represents a promising therapeutic strategy for SS (93). The abnormal expression of IL-17 can run through the entire process of the occurrence and development of SjD and is regarded as an important target for anti-SjD treatment (94). The AKT1/mTOR signaling pathway regulates IL-17 function in a variety of autoimmune diseases (95). miR-let-7d-3p is regarded as the miRNA with the most significant inhibition in expression in patients with SjD (79). Research has found that in SjD patients, the expression of miR-let-7d-3p is negatively correlated with the expression of IL-17. Furthermore, upregulating miR-let-7d-3p expression could modulate the AKT1/mTOR signaling pathway, regulate the proportion of CD4^+^ T cells, reduce IL-17 expression, and alleviate inflammation (96). Previous study has shown that the decreased level of miR-31-5p in CD3^+^T cells of SS patients was closely related to the decreased level of miR-31-5p in CD3^+^T cells of SLE patients (97). Further research has found that by inhibiting the expression of miR-31-5p, the expression of basal extracellular acidification rate (ECAR) and oxygen consumption rate (OCR) of CD4^+^T cells could be reduced, and the OCR/ECAR ratio could be regulated, the glycolytic process could be promoted, the basal level of glucose metabolism could be increased, and ultimately the autoimmune T cell response could be promoted (98).

**Figure 5:**
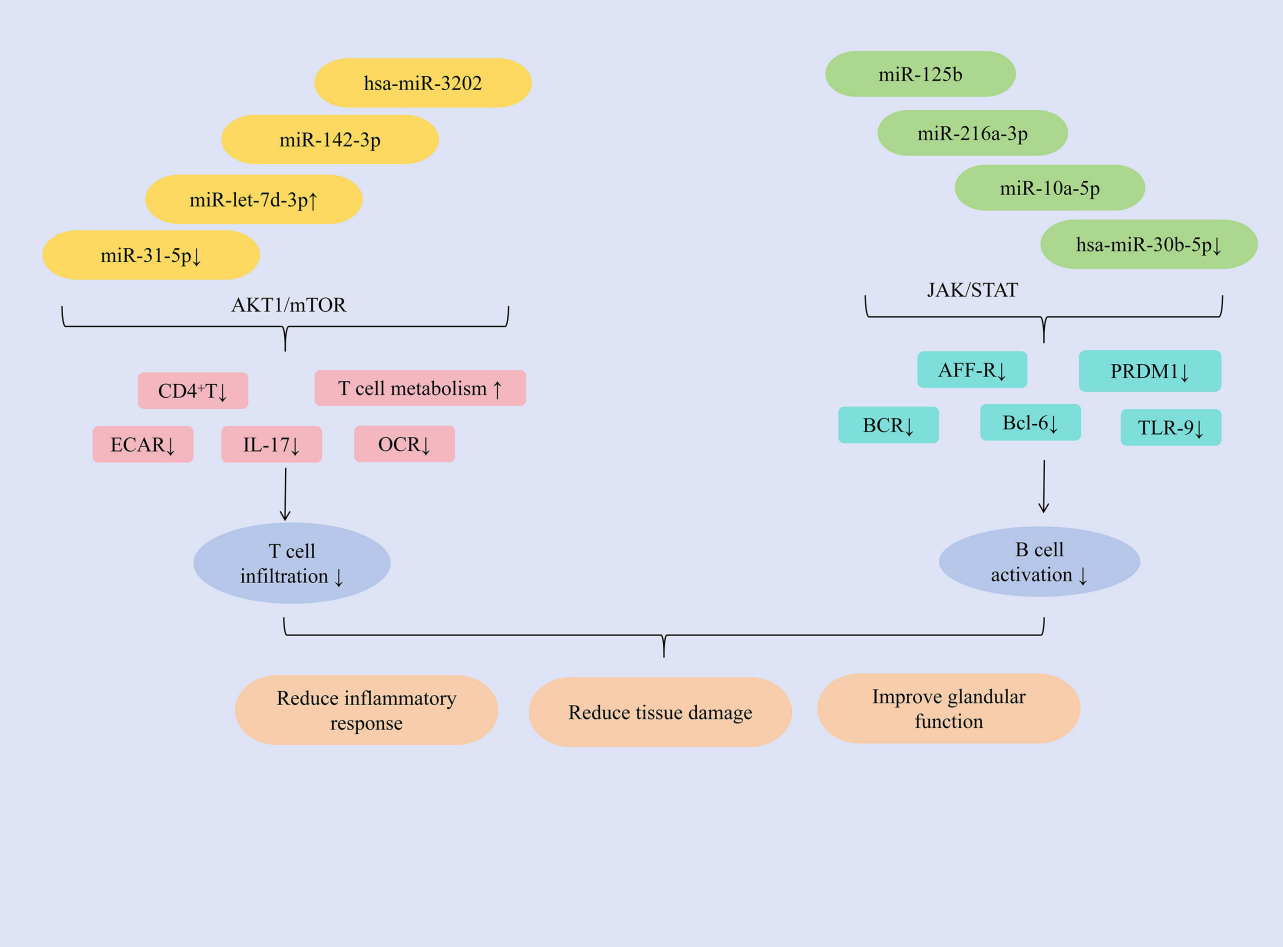
Mechanisms of miRNA regulation of T/B lymphocytes in SjD therapy.

In addition, miR-216a-3p, miR-10a-5p, miR-125b, and the downregulation of hsa-miR-30b-5p expression can inhibit B cell activation, reduce the number and activity of B cells, and thereby alleviate immune infiltration. miRNA expression dysregulation can disrupt immune tolerance and lead to the development of autoimmune diseases (57). Bioinformatic screening of miRNAs targeting STAT1 identified miR-216a-3p as a key regulator. This miRNA is significantly downregulated in SjD and exhibits inverse correlation with STAT1 expression. Functionally, miR-216a-3p downregulates JAK1, JAK2, and STAT1 expression, thereby inhibiting JAK/STAT signaling. Consequently, this reduces protein levels of B-cell activation markers (APR-R, BCR, TLR-9), suppresses B-cell activation, and mitigates immune infiltration. The pathway inhibition concurrently decreases TNF-α, IFN-γ, and IL-6 production–alleviating inflammation and tissue fibrosis. Ultimately, miR-216a-3p restoration restores salivary flow rates and ameliorates xerostomia in SjD rat models (99). Extracellular vesicles derived from myeloid-derived suppressor cells (MDSC-EVs) inhibit autoimmune disease progression by impairing T lymphocyte function (100). Further study has found that miR-10a-5p, delivered by MDSC-EVs, downregulates Bcl-6 expression. This reduces the proportion of germinal center (GC) B cells in experimental Sjögren’s syndrome (ESS) mice, suppresses B cell activation, and consequently improves salivary gland function while significantly inhibiting SS progression (101). Mesenchymal stem cells (MSCs) produce microvesicles and exosomes, which are regarded as the primary bioactive vesicles mediating their paracrine effects. These vesicles serve as effectors of cell signal transduction and intercellular communication, exhibiting significant immunomodulatory functions (102). The study revealed that miR-125b, delivered via labial gland mesenchymal stem cell-derived exosomes (LGMSC-Exos), could reduce PRDM1 (BLIMP-1) expression in B lymphocytes, and inhibit their activation. It decreased the proportion of CD19^+^CD20^+^CD27^+^CD38^+^plasma cells, ameliorated inflammatory responses, and reduced glandular damage in NOD mice (103). B-cell activating factor (BAFF) was upregulated in salivary gland B lymphocytes of patients with SjD. Hsa-miR-30a-3p was found to negatively regulate BAFF expression, likely through binding to the 3′-UTR of BAFF mRNA (104). Study has shown that inhibiting the expression of hsa-miR-30b-5p could significantly increase the expression of BAFF and promote the activation of B cells. Therefore, in summary, BAFF expression is negatively correlated with hsa-miR-30b-5p levels. Increasing hsa-miR-30b-5p expression represents a key strategy to inhibit B cell activation, reduce inflammatory infiltration, and thereby improve glandular function (79). (**Figure 5** presents the regulatory mechanism of miRNAs on T/B lymphocytes in SjD treatment).

### 2.5 miRNA modulation of glandular function

**Figure 6:**
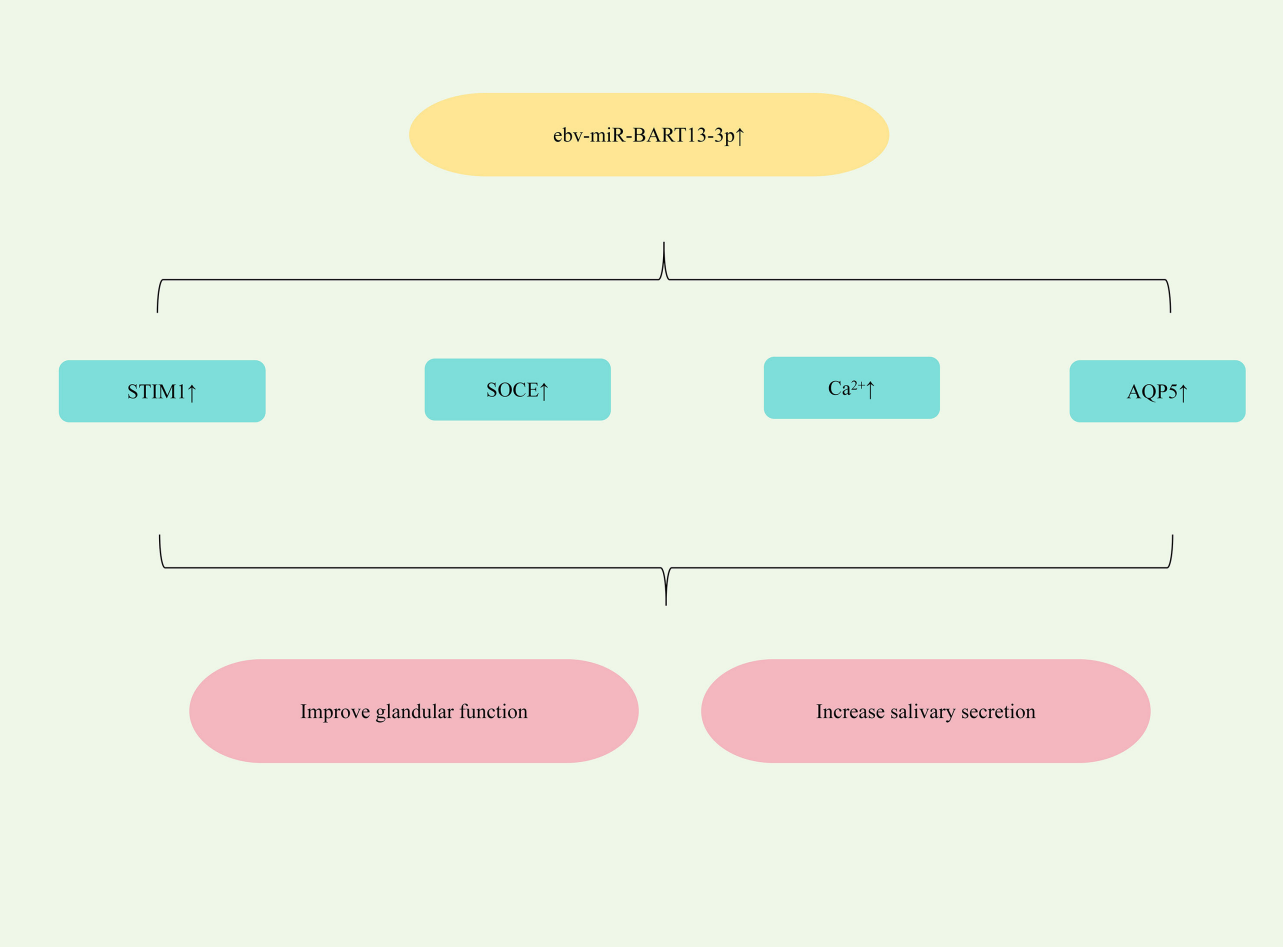
Mechanisms of miRNA-mediated restoration of glandular function in SjD.

All patients with SjD experience glandular dysfunction. This dysfunction arises, in part, from abnormal acinar cell function, which can impair duct cell activation and disrupt progenitor cell homeostasis, ultimately contributing to salivary gland impairment (105). Furthermore, Epstein-Barr virus (EBV) exploits exosomes to transfer its miRNAs from infected B lymphocytes to non-B lymphocytes, promoting salivary dysfunction in SjD (106). Significantly elevated levels of the EBV-specific miRNA ebv-miR-BART13-3p are detected in the salivary glands (SG) of SjD patients (107). Study has found that ebv-miR-BART13-3p could down-regulate the expression of matrix-interacting molecule 1 (STIM1), damage calcium reservoir manipulated calcium entry (SOCE), reduce the expression of Ca^2+^ -dependent genes regulated by SOCE, and thereby impair SG function. It could also down-regulate the expression of AQP5, damage glandular function and reduce saliva secretion (108). Therefore, targeting the upregulation of ebv-miR-BART13-3p represents a potential therapeutic strategy for alleviating dry mouth symptoms in SS patients. (**Figure 6** shows miRNA-mediated restoration of glandular function for SjD therapy).

### 2.6 miRNA regulation of type I interferon response

Study has shown that activation of the type I interferon (IFN) response plays a key role in the pathogenesis of SS (109). The expression of type I IFN response-related proteins in PBMCS derived from SjD patients was significantly increased (110). Crucially, the level of type I IFN response correlates with both glandular and extra-glandular manifestations of SS, and its downregulation can prevent SjD onset and progression (111). Mechanistically, the type I IFN response stimulates excessive B-cell activating factor (BAFF) production in the peripheral blood and salivary glands of SjD patients, thereby promoting B lymphocyte maturation and differentiation (112). Furthermore, this response modulates regulatory T cell (Treg) function, leading to a significantly increased proportion of Treg cells in the serum of type I IFN-positive SjD patients (113). Therefore, type I IFN response is a potential target for the treatment of SjD.

**Figure 7:**
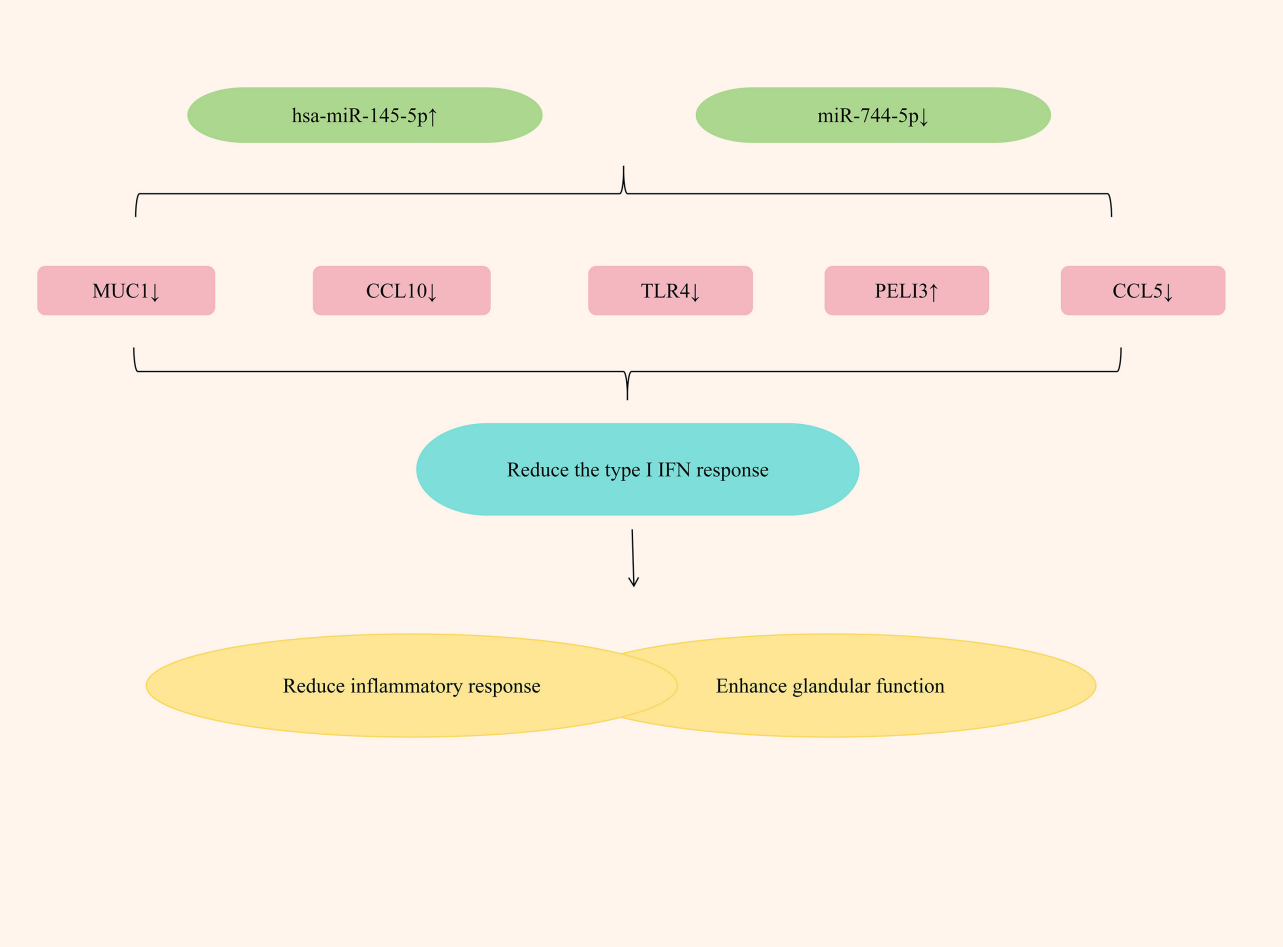
Mechanisms of miRNA regulation of type I interferon response in SjD treatment.

Studies have found that by up-regulating the expression of hsa-miR-145-5p and down-regulating the expression of miR-744-5p, the type I IFN response can be significantly alleviated, exerting an anti-SjD effect. Hsa-miR-145-5p, an anti-inflammatory miRNA, is significantly downregulated in the salivary glands (SG) of SjD patients. This suppression is mediated by type I interferon (IFN), which reduces its expression level (114). Mucin 1 (MUC1) and Toll-like receptor 4 (TLR4) are two related targets of hsa-miR-145-5p. The research has found that in patients with Sjögren’s disease (SjD), by inhibiting the expression of hsa-miR-145-5p, the expressions of MUC1 and TLR4 can be increased, thereby causing salivary gland inflammation and gland dysfunction; if the expression of hsa-miR-145-5p is upregulated, the expressions of MUC1, TLR4 and type I interferons can be reduced, significantly alleviating the inflammatory response, enhancing gland function, and improving dryness symptoms (115). PELI3, a member of the Pellino E3 ubiquitin ligase family, is a recognized negative regulator of inflammation that functions through its interaction with Toll-like receptors (TLRs) (116). In patients with SjD, the expression of miR-744-5p significantly increased, while the expression of PELI3 significantly decreased (48). Further research has found that by down-regulating the expression of miR-744-5p, the expression of PELI3 could be increased, the expression of IFN-dependent chemokines Rantes (CCL5) and CXCL10 could be reduced, the inflammatory response could be significantly alleviated, and the dry eye symptoms of SS patients could be improved (117). (**Figure 7** elucidates miRNA regulation of type I interferon responses in SjD treatment).

### 2.7 miRNA involvement in additional mechanisms

By up-regulating the expression of hsa-miR-181d-5p, the inflammatory response mediated by TNF-α could be significantly alleviated. The up-regulation of miR-130a and miR-708 expression could inhibit the activation of cDC2 cells and alleviate the inflammatory response of SjD.

**Figure 8:**
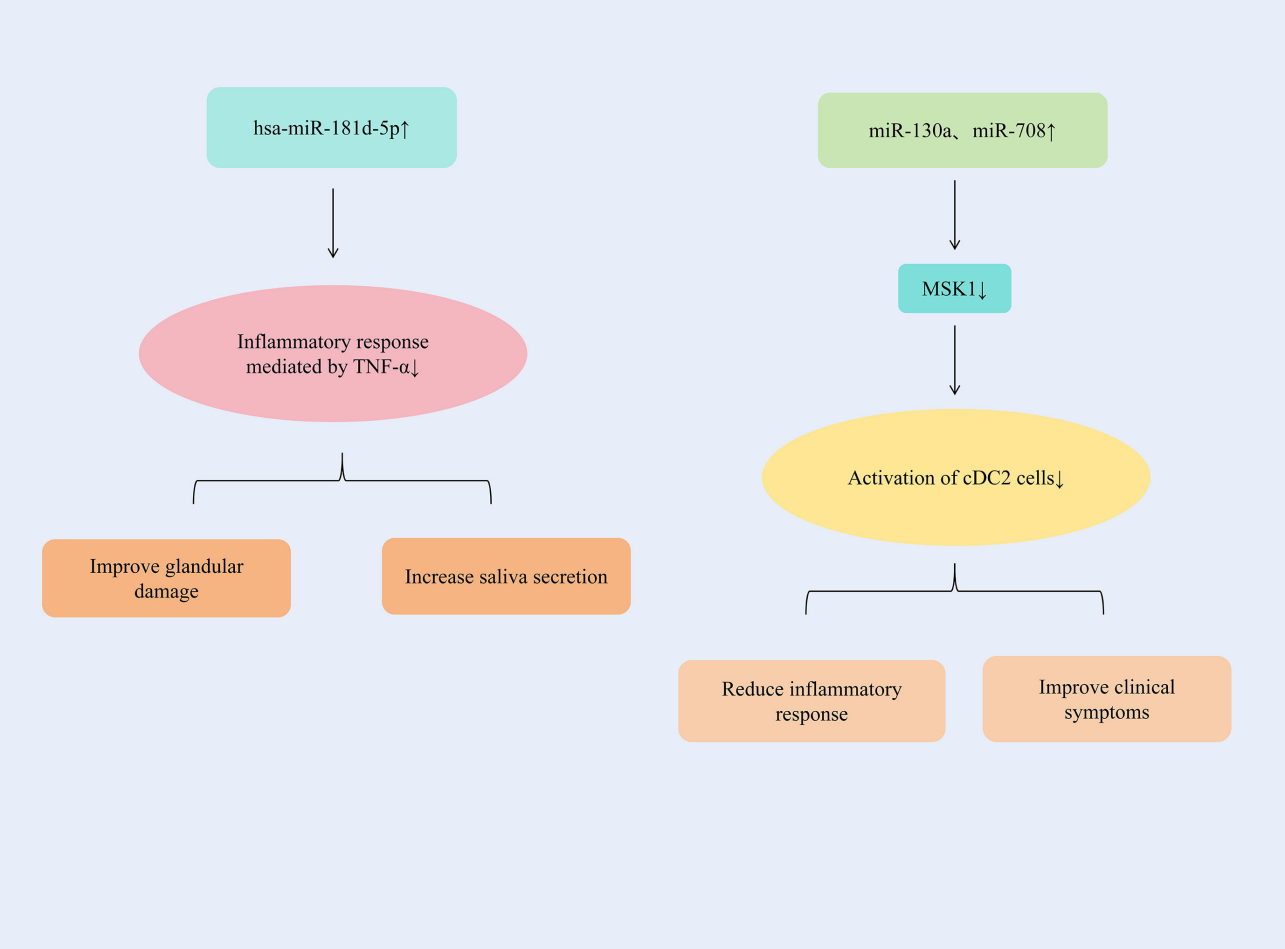
Additional miRNA regulatory mechanisms in SjD therapy.

TNF-α contributes to autoimmune disease progression by facilitating pro-inflammatory cytokine expression, inflammatory cell recruitment, and organ damage. Specifically in the labial salivary glands (LSG) of Sjögren’s syndrome (SS) patients, TNF-α is produced by glandular epithelial cells, infiltrating CD4^+^ T lymphocytes, and monocytes (118). In the miRNA-seq analysis, it was determined that the level of this miRNA had decreased, and one of its main targets was TNFα, which was highly expressed in SS. TNF-α in patients with SjD could increase significantly and was negatively correlated with the presence of hsa-miR-181d-5p. By increasing the expression of hsa-miR-181d-5p, the inflammatory response mediated by TNF-α could be significantly alleviated, glandular damage could be improved, and salivary secretion could be increased (119). Conventional dendritic cells (CDCS) are effective antigen-presenting cells and play a significant role in the initiation and control of immune responses. CDCS could be classified into two subgroups with different phenotypes and functions, namely CD141 (cDC1) and CD1c (cDC2). The main target cells of cDC2, CD4^+^T cells, are considered to play a crucial role in the immunopathology of SjD (120). There was an increased number of DCS in the salivary glands of patients with SjD (121); Therefore, it is suspected that cDC2 plays an important role in driving salivary gland inflammation (122). The expressions of miR-130a and miR-708 continued to decrease in cDC2 of patients with SjD, which might be related to cell activation. MSK1 is an important mediator upstream of NF-κB, controlling cDC2 to produce pro-inflammatory cytokines. By increasing the expressions of miR-130a and miR-708 in cDC2, the expression of MSK1 could be significantly reduced and the activation of cDC2 cells could be inhibited. And it reduced the expressions of TNF-α, IL-6 and IL-12, alleviated the inflammatory response, and improved the symptoms of patients with SjD (123). (**Figure 8** summarizes additional miRNA regulatory mechanisms in SjD therapy.) (**Table 1** summarizes the therapeutic mechanisms of miRNAs in SjD).

**Table 1:** Summary of the mechanism of miRNA in the treatment of SjD.

Regulatory mechanism	miRNA (expression situation)	Animal models/cell models/clinical patients	Regulatory signaling pathways	Specific regulatory effect	Therapeutic action	Ref.
Regulate macrophage polarization	miR-31-5p (↑)	PBMC of patients with SS, rabbits with autoimmune dacryocystitis	p38 MAPK	NOS2, IL-1β, TNF-α↓, Arg-1, CD206, IL-10↑; M1 polarization↓, M2 polarization↑	Inflammatory infiltration, tissue damage, dry eye symptoms↓	(39)
	miR-125b (↓)	SS mice	-	CD38↓, CD206↑, M2 polarization↑	Inflammatory response↓, salivary gland function↑	(42)
	miR-100-5p (↑)	Rabbits with dry eye syndrome	-	NOS2, IRF5↓, CD206, Arg-1, KLF4↑, M2 polarization↑; CD4^+^Foxp3^+^Tregs↑, Tregs differentiation↑; TNF-α, IL-1β, IL-6↓	Inflammatory response, degree of dry eyes↓	(44)
Regulate cell apoptosis	miR-155-5p (↓)	SjD mice, HSGEC cells	PI3K/AKT	Apoptosis↓, Cell viability and colony-forming ability↑; IL-10, IL-4↑, TNF-α, IL-6↓	Inflammatory response↓, glandular secretory function↑	(54)
		SGECs cells	NF-κB	Bax, caspase 3, caspase 9↓, Bcl-2↑, Apoptosis↓; TNF-α, IL-6↓	Inflammatory response, salivary gland damage↓	(58)
	miR-34a(↓)	HSGE cells of SjD patients	NF-κB	Bcl-2↑, pro-caspase 9, caspase 9, cleaved-Caspase-3, Cyt C↓, Apoptosis↓	Salivary gland damage↓	(60)
	miR-223-3p (↓)	SEGCs cells of SjD patients	NF-κB	Bax, caspase-3 ↓, Bcl-2↑, Apoptosis↓; IL-6, IL-12, TNF-γ↓	Inflammatory response↓, tear secretion↑	(61)
	miR-1207-5p,miR-4695-3p(↓)	HSG cells of SjD patients	-	Bax, CASP-9, CASP-8↓, Bcl-2↑, Apoptosis↓	Salivary gland function, salivary secretion↑	(63)
Regulate the balance between Th17/Treg cells	miRNA let-7f-5p(-)	NOD/ShiLtJ mice	RORC/IL-17A	CD4^+^Foxp3^+^↑, CD4^+^IL-17A^+^↓, Th17/Treg balance; IL-10, TGF-β↑, IL-6, IL-17A↓	Salivation↑, lymphocyte infiltration↓	(73)
	miR-29a-3p (-)	NOD, Balb/c mice	miR-29a-3p/T-bet	Th1↓, Th differentiation↓; CD4^+^T↓, IFN-γ, TNF-α↓	Inflammatory response↓, immune microenvironment, salivation↑	(76)
	miR-23b-3p (↑)	NOD mice	NF-κB	TNF-α, IL-6, IL-17A, ING-γ↓, TGF-β1↑, Th1/Th17/Treg balance; Bax, caspase-3↓, Bcl-2↑, Apoptosis↓	Inflammatory response↓, salivation↑	(78)
	miR-146a-5p(↑)	PBMC of patients with SjD	IL23/IL23R	ADAM17, mIL-23R↓, IL-17A, IL-21↑, Th17↑, Th17 differentiation↑	Immuno-inflammatory responses↓	(82)
Regulate T/B lymphocytes	hsa-miR-3202(-)	PBMC of patients with SjD	-	MMP2↓, T cell infiltration, proliferation and invasion↓	Immune inflammatory response, labial gland injury↓, glandular function, salivation↑	(89)
	miR-142-3p (↓)	The salivary glands of patients with SjD	-	cAMP generation↓, calcium signaling↓, protein generation↓; T cell activation↑	Epithelial cell function, glandular function↑	(93)
	miR-let-7d-3p(↑)	PBMC of patients with SjD	AKT1/mTOR	CD4^+^T↓, IL-17↓	Inflammatory response↓	(96)
	miR-31-5p (↓)	PBMC of patients with SjD	-	ECAR, OCR↓, Glucose metabolism↑, T-cell response↑	Inflammatory response↓	(98)
	miR-216a-3p(-)	SjD rats	JAK/STAT	AFF-R, BCR, TLR-9↓,B cells differentiation↓; TNF-α, IFN-γ, IL-6↓	Inflammatory response, tissue fibrosis↓, salivation↑	(99)
	miR-10a-5p (-)	ESS mice	-	Bcl-6↓, The number and activation of B cells↓	Salivary gland function↑	(101)
	miR-125b(-)	The labial glands of patients with SjD, NOD mice	-	PRDM1↓, B cell activation↓; The proportion of CD19, CD20, CD27 and CD38 cells↓	Inflammatory response, glandular damage↓, salivary flow rate, salivary secretion↑	(103)
	hsa-miR-30b-5p(↑)	PBMC of patients with SjD	-	B cell activation↓	Inflammatory infiltration↓, glandular function↑	(79)
Regulate glandular function	ebv-miR-BART13-3p(↓)	The salivary glands of patients with SjD	-	STIM1, SOCE, Calcium-dependent gene, AQP5↑	Glandular function, salivary secretion↑	(108)
Regulate the type I IFN responses	hsa-miR-145-5p(↑)	The salivary glands of patients with SjD, HSG cells	-	MUC1, TLR4, Type I IFN↓	Inflammatory response↓, glandular function↑	(115)
	miR-744-5p (↓)	The salivary glands and PBMC of patients with SjD	-	PELI3↑, CCL5, CXCL10↓	Inflammatory response↓	(117)
Regulate other mechanisms	hsa-miR-181d-5p(↑)	The salivary glands of patients with SjD	-	TNF-α mediates inflammatory responses↓	Glandular damage↓, salivation↑	(119)
	miR-130a, miR-708(↑)	PBMC of patients with SjD	-	MSK1↓, cDC2↓, TNF-α, IL-6, IL-12↓	Inflammatory response↓	(123)

↑: Enhance/activate; ↓: Reduce/inhibit.

## 3 Discussion

miRNAs critically regulate the pathogenesis, progression, and prognosis of primary Sjögren’s disease (SjD). As a diagnostic biomarker for pSS, miRNAs possess the characteristics of tissue specificity, high stability, and ease of detection. For instance, miR-17-5p, miR-146a-5p and miR-155-5p, etc. show highly specific expression in the serum of patients with pSS and are related to the disease activity. They are expected to become highly specific diagnostic markers. However, their clinical application still needs to address issues such as standardization, validation and cost. In pSS, miRNAs interact with lncRNAs (such as XIST) and circRNAs through the ceRNA mechanism, and target epigenetic modification enzymes (such as miR-29 inhibiting DNMTs). They jointly form a regulatory network that affects DNA methylation, NF-κB pathways, breaks immune tolerance, promotes lymphocyte infiltration and glandular damage, and drives disease progression.

Summary and analysis are as follows: 1) signaling pathways: miRNAs predominantly modulate the NF-κB, JAK/STAT, and PI3K/AKT pathways. For example, downregulation of miR-155-5p, miR-223-3p, and miR-34a, or upregulation of miR-23b-3p, inhibits NF-κB signaling; 2) mechanistic targets: key regulatory functions include macrophage polarization, apoptosis, Th17/Treg balance, T/B lymphocyte activity, glandular function, and type I interferon (IFN) responses; 3) expression dynamics: most studies report directional miRNA alterations (up-/down-regulation). Apoptosis is typically regulated through miRNA downregulation, while macrophage polarization, T/B lymphocytes, and type I IFN responses exhibit bidirectional miRNA regulation. However, some studies lack explicit directionality; 4) research models: clinical therapeutic potential is demonstrated for miRNAs like miR-223-3p and miR-125b, while other evidence derives from *in vivo* (NOD mice) or *in vitro* (e.g., SGECs) models; 5) Certain miRNAs exhibit multi-mechanistic regulatory roles in SjD pathogenesis. For instance: miR-125b modulated macrophage polarization and promotes Th17 activation; miR-100-5p regulated macrophage polarization while enhancing Treg activation; miR-23b-3p balanced Th17/Treg ratios and inhibits apoptosis. Notably, individual miRNAs could target distinct signaling pathways. For example, down-regulation of miR-155-5p can inhibit apoptosis by regulating the PI3K/AKT and NF-κB signaling pathways respectively.

However, several deficiencies persist in current research: 1) Some studies have not fully elucidated the specific signaling pathways involved in the role of miRNA. For instance, miR-125b, miR-100-5p, miR-1207-5p, etc. still lack the exploration and clarification of signaling pathways. This might be determined by the inherent multi-target characteristics of miRNAs, the complexity of the cross-interaction of signaling pathways, the difficulty in validating the targets, and the limitations of the SjD disease model. 2) The regulatory role of miRNA expression itself remains unclear. For instance, miR-29a-3p, hsa-miR-3202, miR-216a-3p, etc. lack regulatory effects. This might be related to the influence of transcription factors, epigenetics and other factors on the abnormal expression of specific miRNAs in SjD, and further research and clarification are needed. 3) The interactions of miRNA in the pathogenesis of SjD and their common and unique roles are not yet fully understood. For instance, miR-1207-5p and miR-4695-3p jointly regulate cell apoptosis, while miR-130a and miR-708 jointly regulate cDC2. However, it is unclear how they interact, cooperate or antagonize to regulate the pathological and physiological processes of SjD. Further research is needed to strengthen this understanding. 4) Various miRNAs such as miR-125b, miR-100-5p, and miR-23b-3p possess multi-target and multi-pathway regulatory properties. They can regulate macrophage polarization, cell apoptosis, and Treg differentiation, etc. However, the relative importance and causal relationship of these miRNAs in different mechanisms of SjD pathology remain unclear and require further research and clarification. 5). Therapeutic strategies based on miRNAs (such as agomiR, antagomiR, viral vectors, and EV delivery) all face challenges in terms of delivery efficiency, safety, and production. Their multi-target characteristics lead to high off-target risks. Although theoretically a miRNA can target hundreds of mRNAs, in the actual cellular environment, its regulatory effect is selective and is mainly influenced by factors such as binding affinity, target abundance, and the expression level of the miRNA itself. The mimics may excessively inhibit unintended targets, while the antagonists may abnormally activate pathways, causing severe adverse reactions, resulting in the termination of clinical trials such as MRX34 and Miravirsen.

Therefore, future efforts should focus on: 1) Advancing miRNA extraction and utilization technologies to enhance safety and maturity; 2) Expanding research on miRNA-related signaling pathways; 3) Validating and elucidating miRNA regulatory mechanisms; 4) Deepening investigations into miRNA interrelationships and their regulatory roles in SjD; 5) Conducting large-scale, rigorous clinical research aligned with clinical needs. Concurrently, clinical translation frameworks for miRNA-based SjD therapies should be strengthened to accelerate novel drug R&D. Emphasis must be placed on improving targeting precision to ensure miRNAs directly engage SjD pathological tissues and maximize therapeutic efficacy.
